# Photocatalytic properties of PbS/graphene oxide/polyaniline electrode for hydrogen generation

**DOI:** 10.1038/s41598-017-14582-8

**Published:** 2017-10-26

**Authors:** Mohamed Shaban, Mohamed Rabia, Asmaa M. Abd El-Sayed, Aya Ahmed, Somaya Sayed

**Affiliations:** 1 Nanophotonics and Applications (NPA) Lab, Department of Physics, Faculty of Science, Beni -Suef University, Beni-Suef, 62514 Egypt; 2 Polymer Research Laboratory, Chemistry Department, Faculty of Science, Beni-Suef University, Beni-Suef, 62514 Egypt

## Abstract

In this work, roll-graphene oxide (Ro-GO), polyaniline (PANI) nano/microparticles, and PbS nanoparticles were prepared by modified Hammer, oxidative polymerization, and chemical bath deposition methods, respectively. These nano/microstructures were characterized, optimized, and designed to form PbS/Ro-GO/PANI nano/microcomposite. Also, the ratios of PbS and Ro-GO were optimized, and the optimized composition of the used composite was 0.4 g PANI, 0.125 g Ro-GO, and 0.075 g PbS. The band gap values for PANI, PbS, Ro-GO, and PbS/Ro-GO/PANI rocomposite were 3, 1.13, 2.86, (1.16, 2) eV, respectively. Two photoelectrode assemblies, Au/PbS/Ro-GO/PANI and PbS/Ro-GO/PANI/ITO/glass were used for the photoelectrochemical (PEC) hydrogen generation. In the first assembly 45 nm- Au layer was sputtered on the surface of a disk of PbS/Ro-GO/PANI composite. For the second assembly, a disk of PbS/Ro-GO/PANI composite was glued on ITO glass using Ag-THF paste. The lifetime efficiency values were 64.2 and 43.4% for the first and second electrode for 2 h, respectively. Finally, the incident photon-to-current conversion efficiency (IPCE) and photon-to-current efficiency (ABPE) were calculated under monochromatic illumination conditions. The optimum IPCE efficiency at 390 nm was 9.4% and 16.17%, whereas ABPE % efficiency was 1.01% and 1.75% for Au/PbS/Ro-GO/PANI and PbS/Ro-GO/PANI/ITO/glass, respectively.

## Introduction

To date, the global electricity generation capacity has been estimated to exceed 20 terawatt hours and more than 70% of that electrical energy supply is from fossil fuels. Unfortunately, the production of fossil energies like oil and natural gas cannot meet the increasing global energy demand in the near future. Fossil fuels are the major energy sources that lead to disastrous effects such as air, water, and soil pollutions. The burning of varies fossil fuels releases carbon dioxide, nitrogen monoxide, nitrogen dioxide, sulfur dioxide, and carbon monoxide. They have severe consequences on the habitats and the human health^1^.

Recently, the developing of new energy sources to replace the traditional ones become critical. Hydrogen as a kind of renewable energy has attracted more attention due to its pollution-free, low cost, high combustion power, and high efficiency^2,3^. The production of hydrogen gas was carried out using chemicals, photoelectrochemical, and electrochemical methods. The photocatalytic water splitting for H_2_ generation utilizing semiconductor nanostructure and sunlight is considered an important source of renewable energy^4^.

In 1972 Fujishima and Honda *et al*. used photo-electrochemical method for splitting water into hydrogen and oxygen using TiO_2_ as an electrode^5^. Despite the successful production of H_2_, the splitting occurred using UV light not the visible light; this was due to the large band gap of TiO_2_ material (3.2 eV)^6^. Since then, semiconductors have widely used as catalysts for photocatalytic hydrogen production. To utilize the visible light, 43% of the solar spectrum, the band gap must be in the range 1.7–2.2 eV. This value may be in the range of many other semiconductor materials, including oxides, oxynitrides, and oxysulfides^7^.

Graphene is an attractive 2D layered hexagonal lattice of carbon nanomaterial with atomic thickness. Graphene possesses superior transport and electronic properties^8^. Graphene oxide (GO) is an oxidizing form of graphene. GO is not a good conductor but a reduction process can restore the graphene structure and conductivity. GO is one of the most intensively studied nanomaterials which can be utilized for various potential applications such as solar cells, hydrogen storage, batteries, catalysts, and sensors^9,10^. Therefore, there were some trials to change the topological structure of GO to obtain enhanced properties and some different performances^11^. With the aid of sonication, Viculis *et al*. produced carbon nanoscroll using graphite intercalation compound (KC_8_)^12^. Also, Savoskin *et al*. studied the production of carbon nanoscroll from acceptor-type graphite intercalation compounds under sonication effects^13^. Moreover, Loh *et al*. transformed 2D GO nanosheets into carbon nanotubes by sonicating GO in 70% nitric acid^14^.

On the other hand, conducting polymers gained a considerable interest because of their electronic, magnetic, and optical properties^15,16^. As a conventional conducting polymer, polyaniline (PANI) is cheaper than other conducting polymers and has excellent processability, environmental stability, and photoelectric property^17^. Composite of PANI integrated carbon material, metal oxide, or sulfide display significantly enhanced conductivity and electrocatalytic activity. Various electrical and electrochemical systems or devices have been designed based on polymer/metal oxide or metal sulfide composites^18^. Hence, it is expected that the combination of GO, PANI, and PbS nanomaterials could improve the structural, optical, electrical, and photocatalytic properties^19^. Chou *et al*. studied the preparation and characterization of bimetallic Ni/Co/GO nanoparticle catalysts for H_2_ generation from catalytic hydrolysis of sodium borohydride in the presence of NaOH^20^. Shi *et al*. studied the multiple exciton generations using ZnO/PbS/GO photocatalyst for H_2_ production from the water^21^. The existence of multiple exciton generation processes in PbS improved the photocatalytic efficiency of hydrogen production combined with the electron-hole separation of GO. Nsib *et al*. synthesized Ni/Zn/PANI hybrid photocatalysts for hydrogen production from water splitting under visible irradiation^22^. Zhang *et al*. prepared MoS_2_/PANI with abundant protonated sites for electrochemical hydrogen evolution using 0.5 M H_2_SO_4_ as a hydrogen source. The hydrogen generation performance has been achieved with low onset potential of 100 mV and a Tafel slope of 45 mV dec^−1^ ^23^.

Nobel metals such as Pt, Ru, and Rh demonstrate highly activities towards the hydrolytic dehydrogenation^24^. The synergistic effect of Au has generated enormous scientific interests for surface protection and improvement the catalytic activity by subtly adjusting the electronic state and accelerating the electron transfer^25^. Although the previous studies attempted to produce photo nanocomposites for an efficient photoelectrochemical hydrogen generation as a source of renewable energy, however, the hydrogen production efficiency and the stability of the photoelectrode still low. Xiaoa *et al*. prepared WS_2_/poly(3,4-ethylene dioxythiophene)/Au composites electrode for the H_2_ evolution reaction with J_ph_ values of 1.4–2.6 mA.cm^−2^, but they did not determine IPCE or ABPE efficiency^26^. By the same manner, Ramohlola *et al*. prepared poly(3-aminobenzoic acid)/organic framework electrode with maximum J_ph_ of 0.13 mA.cm^−2^ using H_2_SO_4_ electrolyte^27^. Moreover, Jin *et al*. prepared inorganic TiO_2_/CdS/PbS composite electrode for H_2_O splitting with IPCE efficiency of 4% and J_ph_ value of 2 mA.cm^−2^ in the electrolyte of Na_2_S/Na_2_S_2_O_3_^28^. Jin-Nouchi used PbS/TiO_2_ electrode with IPCE efficiency of 13% in the presence of Na_2_S/Na_2_S_2_O_3_ electrolyte^29^_._ Also, Thimsen *et al*. studied the influence of plasmonic Au nanoparticles on the photoactivity of Fe_2_O_3_ electrodes for water splitting with ABPE efficiency of 1% and J_ph_ value of 1.2 mA.cm^−2^ using NaOH as an electrolyte^30^. There are some limitations of some previous studies such as difficulty and high-cost techniques used in the preparation of photo nanocomposites such as pulsed laser deposition and electrodeposition station^22,23^.

This work aims to prepare two different configurations based on PbS/RoGO/PANI nano/microcomposite for the efficient photoelectrochemical generation of H_2_ under visible irradiations. The ratios of PbS and Ro-GO in the nano/microcomposite are optimized. The morphological, structural and optical properties of the studied structures are addressed. The photoelectrochemical behaviors; current-voltage (I-V) and the current time (I-t); are measured. Finally, IPCE and ABPE efficiency under monochromatic illumination conditions were calculated.

## Results and Discussion

### Characterization of the prepared nano/microstructures

It is well known that the morphological and structural properties of nano/microstructures are strongly influenced their optical and photoelectrochemical properties. For this reason, it is crucial to investigate the structural and morphological properties of the PANI, Ro-GO, PbS, and PbS/Ro-GO/PANI nano/microstructures.

**Figure 1:**
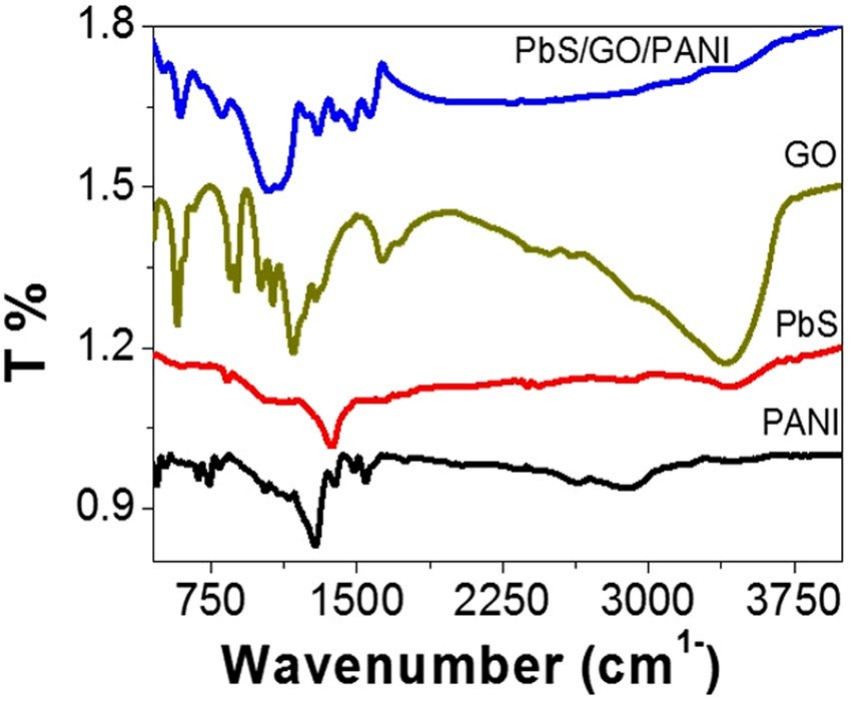
FTIR spectra of PANI, PbS, Ro-GO, and PbS/Ro-GO/PANI nano/microstructures at 298 K.

Figure 1 shows the FT-IR spectra of the studied structures. Also, the positions of the major peaks of PANI, Ro-GO, PbS, and PbS/Ro-GO/PANI nano/microstructures and their assignments are illustrated in Table 1. From Fig. 1 and Table 1, there are small redshifts of bands of PbS and Ro-GO nano/microparticles in comparison with that observed for the PbS/Ro-GO/PANI composite. This appears in the frequencies of heteropolar diatomic molecules of PbS nanoparticles and the C–O epoxide group stretching in Ro-GO particle. Also, there are blue-shifts in some groups of the benzenoid or quinoid rings of the composite in comparison with the PANI alone. These shifts clearly appear in the C=C stretching and C-H in-plane bending vibrations, in addition to the chloride group incorporation in the composite. These shifts are a result of the interaction between the constituents of the formed composite. The other bands for all components have almost the same values before or after the formation of nano/microcomposite.

**Table 1:** FTIR analyses of the prepared nano/microstructures at 298 K.

Band position (cm^−1^)				Assignment
PbS/Ro-GO/PANI	PANI	PbS	Ro-GO	
3400	3401	—	3400	O–H stretching vibrations −OH groups in Ro-GO. N–H stretching vibrations of amino groups in PANI^31,32^
2914	2918	—	—	Vibration of C-H aromatic ring
1566	1561	—	1632	The coordinated water molecule^33,34^
1480	1467	—	—	C=C stretching vibrations of quinoid ring^35^
1391	—	1400	—	Frequency of heteropolar diatomic molecules of PbS^36^
1301	1301	—	—	C=C vibration of benzenoid rings
1101	—	—	1155	the C–O epoxide group stretching^37^
1101	1105	—	—	C–N stretching vibrations
1044	—	1061	—	Frequency of heteropolar diatomic molecules of PbS^36^
1044	1015	—	—	Chloride group incorporation in the polymer chain^37^
808	789	—	—	C–H in-plane bending vibration^38,39^
593	587	—	—	Para disubstituted aromatic rings

**Figure 2:**
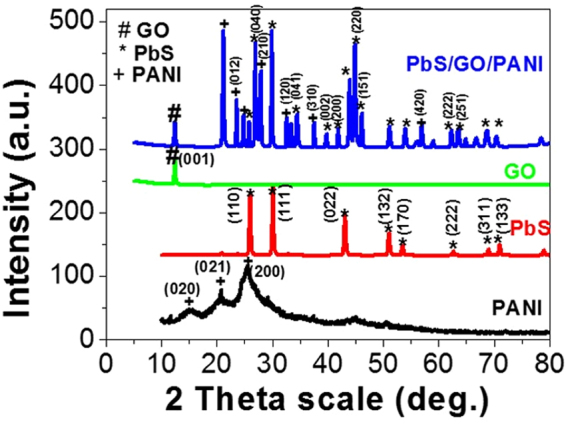
XRD spectra of PANI (black line), PbS (red line), Ro-GO (green line), and 0.075 g PbS/0.125 g Ro-GO/0.4 g PANI nano/microcomposite (blue line) at 298 K.

The XRD spectra for PANI, PbS, Ro-GO nano/microparticles, and PbS/Ro-GO/PANI nano/microcomposite are shown in Fig. 2. The XRD spectrum of PANI (black-line) clearly indicated the preparation of PANI crystallites with crystalline domains. Three distinct crystalline peaks appeared centered at 2θ = 15.18°, 21.12°, and 25.49°, which corresponding to (020), (021), and (200) crystal planes, respectively, of PANI in its emeraldine salt form^40^. The characteristic peaks at 2θ = 15.18° and 25.49° are ascribed to the perpendicular and parallel periodicity of the polymer chain, respectively^41,42^. The average size of the PANI crystallites is determined from the full width at half maximum (W) in radians using Scherrer’s formula; D = 0.9λ/W cos θ; where λ is the X-ray wavelength (CuKα = 0.15405 nm)^43^. The calculated value of the average crystallite size of the PANI is ~100 nm.

As shown in the XRD spectrum of PbS (red line), there are eight distinct crystalline peaks centered at 2θ = 25.98°, 30.17°, 43.12°, 51.14°,53.70°, 62.0°, 68.82°, and 71.01° corresponding to (110), (111), (022), (132), (170), (222), (311), and (133) orientations, respectively. The average size of the PbS crystallite is ~36.3 nm. The XRD of Ro-GO (green line), shows only one peak at 2θ = 10° in the direction (001). The average size of Ro-GO crystallites is about 117 nm.

Finally, the characteristic peaks of PbS, Ro-GO, and PANI appear the XRD pattern of (0.075 g) PbS/(0.125 g) Ro-GO/(0.4 g) PANI nano/microcomposite with slight right shifts in the peaks because of composite formation^44^. In addition, there are new peaks appeared in the XRD pattern of the composite. This pattern shows five peaks centered at 2θ = 23.3°, 28°, 32.4°, 37.5°, 57.0° for PANI nanocrystallites grown along (012), (210), (120), (310), and (420) orientations, respectively. Also, it shows six distinct crystalline peaks centered at 2θ = 27.7°, 39.6°, 41.8°, 44.3°, 45.9°, and 63.2° for PbS grown along (040), (002), (200), (220), (151), and (251) orientations, respectively. The new peaks appeared for both PbS and PANI in the formed composite is ascribed to the interaction and interference between the constituents of the composite. The average crystal size of the composite that calculated using Scherrer’s formula is 47 nm.

**Figure 3:**
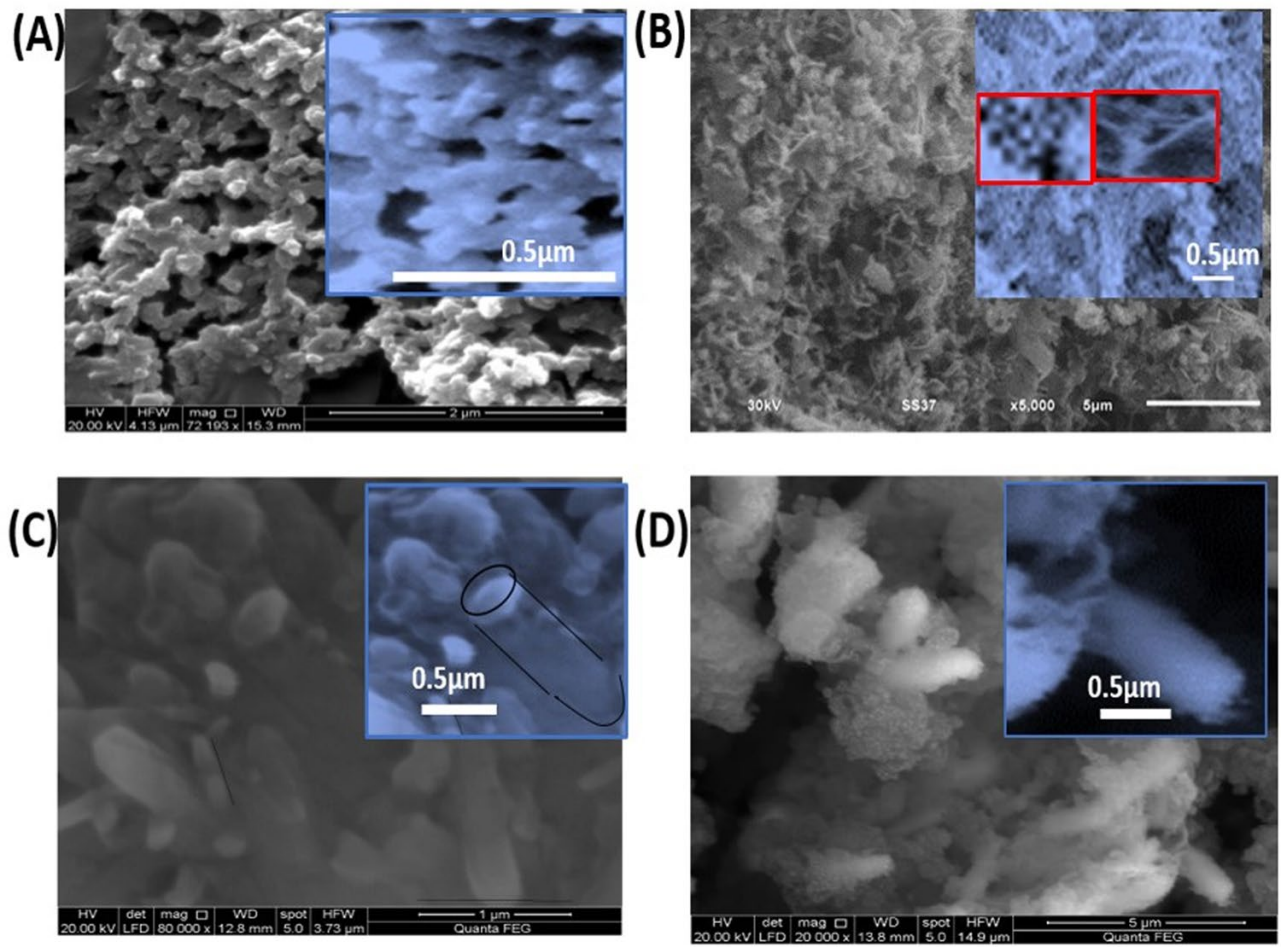
SEM images of (**A**) PANI, (**B**) PbS, (**C**) Ro-GO, and (**D**) PbS/Ro- GO/PANI nano/microcomposite.

The surface morphology of the prepared PANI, PbS, Ro-GO, PbS/Ro-GO/PANI nano/microcomposite are examined by SEM as shown in Fig. 3. SEM image of PANI, Fig. 3(A), illustrates the fabrication of nano/microparticles PANI network with average particle size of 180 nm. Figure 3(B) demonstrates the fabrication of PbS nanostructure from mixed nanofibers and nanoparticles. The average particles size is 50 nm. As shown in the inset, these nanoparticles are agglomerated and self-assembly to show nanoporous regimes. Figure 3(C) illustrates the fabrication of rolled GO (Ro-GO) with an average diameter of 140 nm. This indicates the rolling of some flat layers of GO after preparations to form roles. Finally, Fig. 3(D) shows the fabrication of nano/microcomposite (PANI + PbS + Ro-GO) of different shapes. This figure clearly illustrates the complete filling of the Ro-GO and decoration of the whole surface with very fine nano/microparticles as shown in the inset.

**Figure 4:**
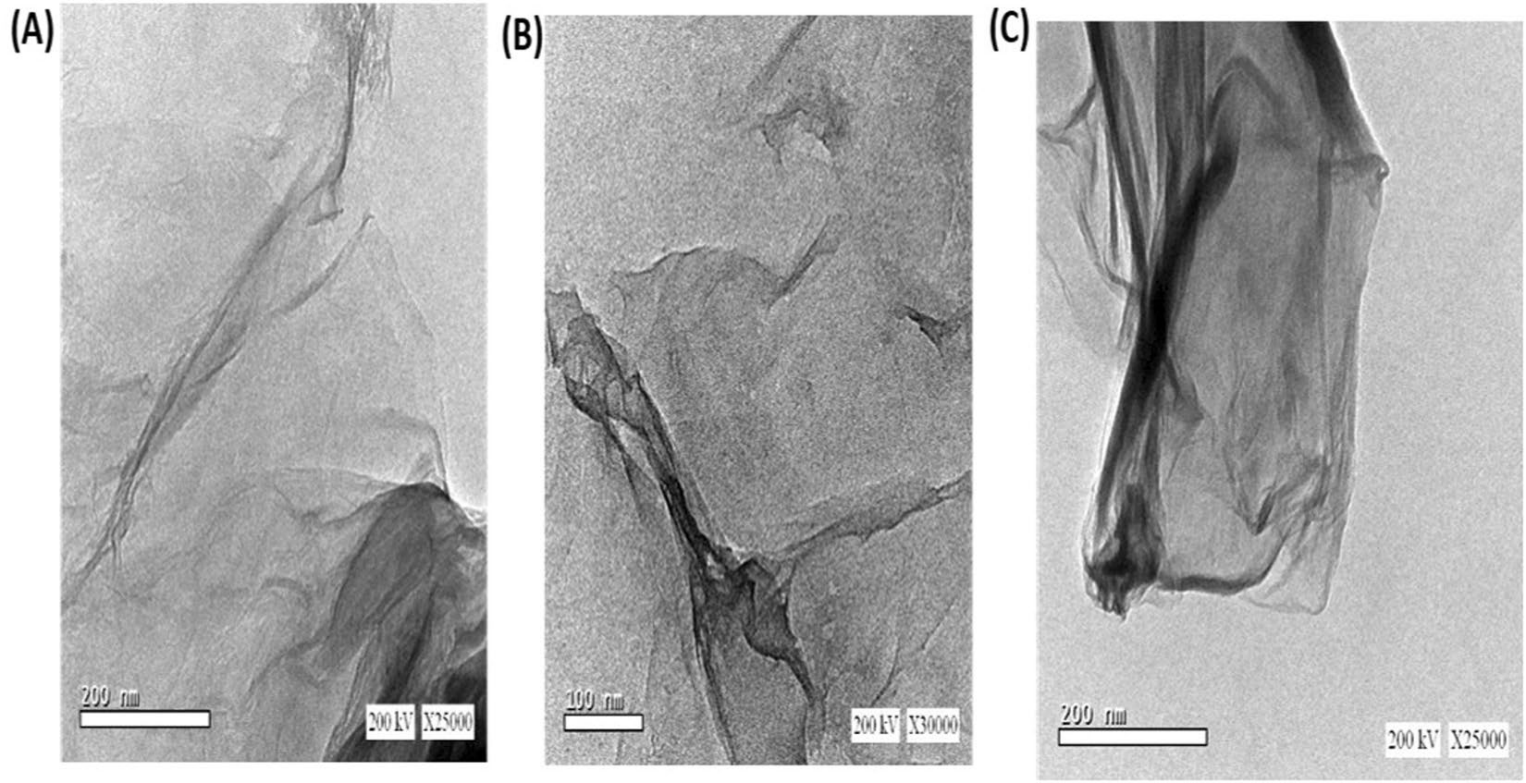
TEM images of Ro-GO nano/microparticles after settling down preparation times (**A**) 10, (**B**) 30, and (**C**) 60 days.

Figure 4(A‒C) shows TEM images for prepared Ro-GO after settling down preparation times varied from 10 to 60 days. From the TEM images, the flat sheet of the prepared GO is rolling up with times to form single or multiwalled Ro-GO. The first rolled sheet acts as nuclei for the other sheets to roll around it with times. The diameter and length of Ro-GO increase with time. The thickness of its wall increases with time due to the multi-rolling process that forms more layers. The average inner diameter increases from 20 to 50 nm with increasing of the settling down times from 10 to 60 days, respectively. Moreover, the outer diameter is increased from 30 to 70 nm with increasing the time.

**Figure 5:**
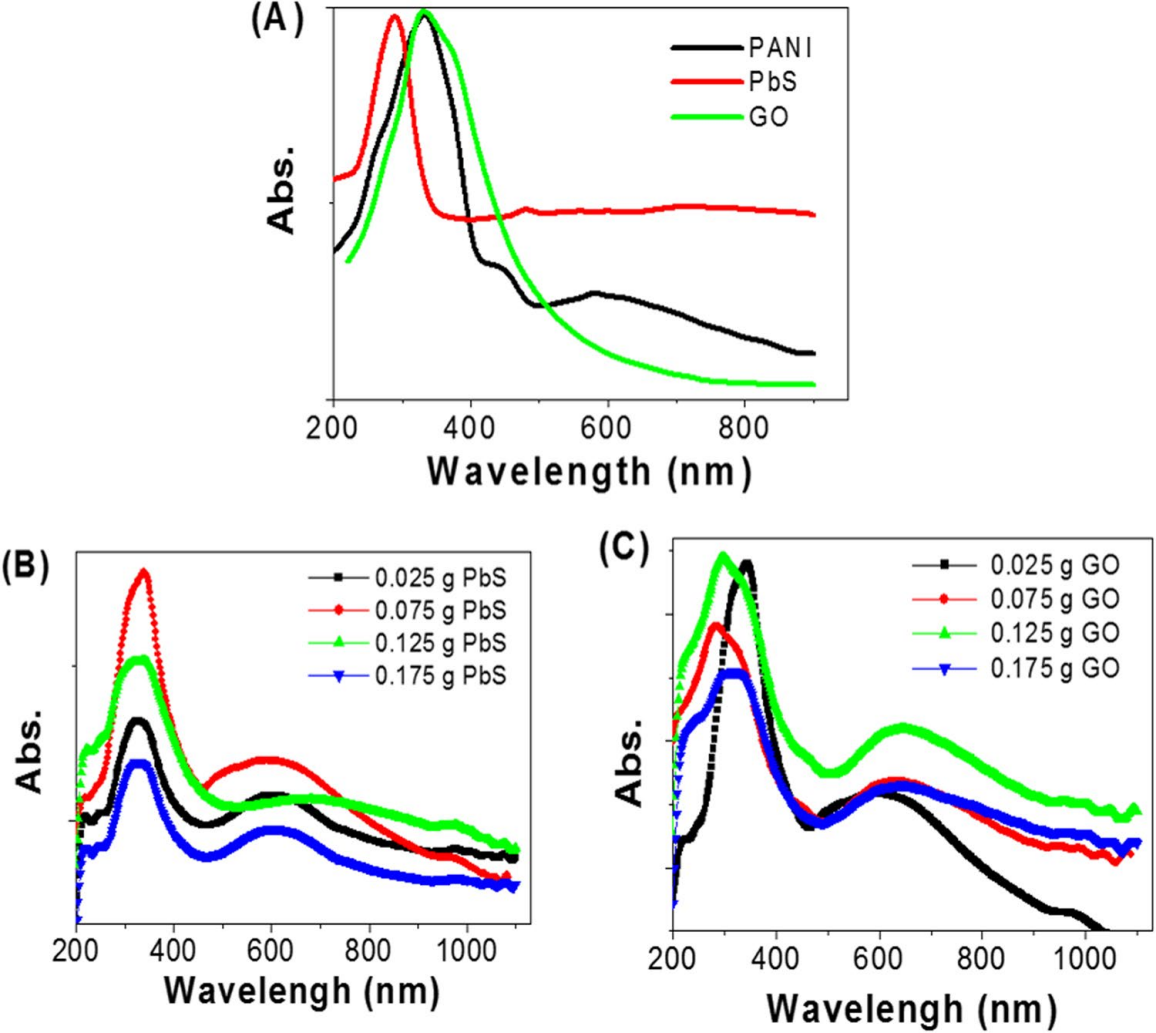
(**A**) UV-Vis spectra for PANI (black line), PbS (red line), Ro-GO (green line) nano/microstructures. Spectra of PbS/Ro-GO/PANI nano/microcomposites with different weight portions of (**B**) PbS and (**C**) Ro-GO.

The study of optical properties of the PANI, PbS, Ro-GO, PbS/Ro-GO/PANI nano/microstructures is a vital factor for the application of these nanostructures in the photoelectrochemical water splitting systems. Figure 5(A) shows the absorption spectra of PANI, PbS, and Ro-GO. The spectrum of PANI, black line, shows one semi-sharp absorption peak at 333 nm in the UV region. Additionally, two broad peaks were observed at 439 and 600 nm in the visible region. The sharp peak is due to Π-Π* transitions from the benzenoid ring^45^, whereas the two broad peaks are due to high conjugation of the aromatic polymer chain^46^.

From the UV–Vis absorption spectrum of PbS nanostructure (red line), there are two characteristic absorption peaks. The sharp one at 296 nm in the UV region, while the another one is a broad peak centered at ~700 nm in the Vis/near IR region. Also, The UV-Vis absorption spectrum of Ro-GO (green line) clearly shows vigorous and wide absorption peak centered at 335 nm. This peak is attributed to the *π*-π* transition of the aromatic C–C ring and *n*-π* transition of C=O bond^46^. The absorption of the Ro-GO has decreased exponentially for λ ≥ 450 nm, and the absorption is very close to zero for IR range.

The optimum ratios PbS and Ro-GO for the preparation of optimized PbS/Ro-GO/PANI composite are studied using the optical analyses as shown in Fig. 5(B,C). The composite has two broad absorbance peaks at about 330 and 620 nm as shown in Fig. 5(B,C). The position and intensity of these two peaks are strongly affected by the weight portions of the PbS and Ro-GO in the composite. As shown in Fig. 5(B), by increasing the weight portion of PbS to 0.075 g, the intensity of the absorption peaks increased and then decreased with further increase of the PbS weight portion to 0.175 g in the composite. These optical spectra clearly indicate that 0.075 g of PbS is the most suitable weight portion for the fabrication of optimized photoelectrode for PEC experiment.

The effect of Ro-GO weight portion variation from 0.025 to 0.175 g on the optical spectra of the composite is shown in Fig. 5(C). From the figure, the absorbance values increase with increasing Ro-GO weight portion from 0.025 to 0.125 g, then decrease with further increasing of Ro-GO weight. Additionally, the UV-Vis peaks positions are redshifted by increasing of Ro-GO weight portions. This appears in the increasing weight portion from 0.075 to 0.125 g, in which the UV peak position increased from 290 to 300 nm, respectively, and the Vis peak position increased from 635 to 650 nm, respectively. So, the optimum composition of nano/microcomposite is 0.4 g PANI + 0.075 PbS + 0.125 g Ro-GO.

Based on direct allowed transition type, the optical band gap of all samples is estimated using Tauc’s equation (eq. 1)^47^;

1 $$\alpha ={\rm{A}}{(hv-{E}_{g})}^{1/2}/hv$$

Where 𝛼 is the absorption coefficient, A is the absorbance of the sample, *E*_*g*_ is the optical band gap, h is the Planck constant, *v* is reciprocal of the wavelength. The absorption coefficient is given by^48,49^:

2 $$\alpha =2.303\,\times 103\,A\,\beta /l{\rm{C}}$$

Where β is the density of nano/micromaterials, which are 1.36, 7.6, 0.3, 1.91 g/cm^3^ for PANI, PbS, Ro-GO, and PbS/Ro-GO/PANI (0.4 g PANI, 0.075 g PbS, and 0.125 g Ro-GO), respectively. L is the path of the quartz cell (1.0 cm), and C is the concentration of the powder in the suspension.

**Figure 6:**
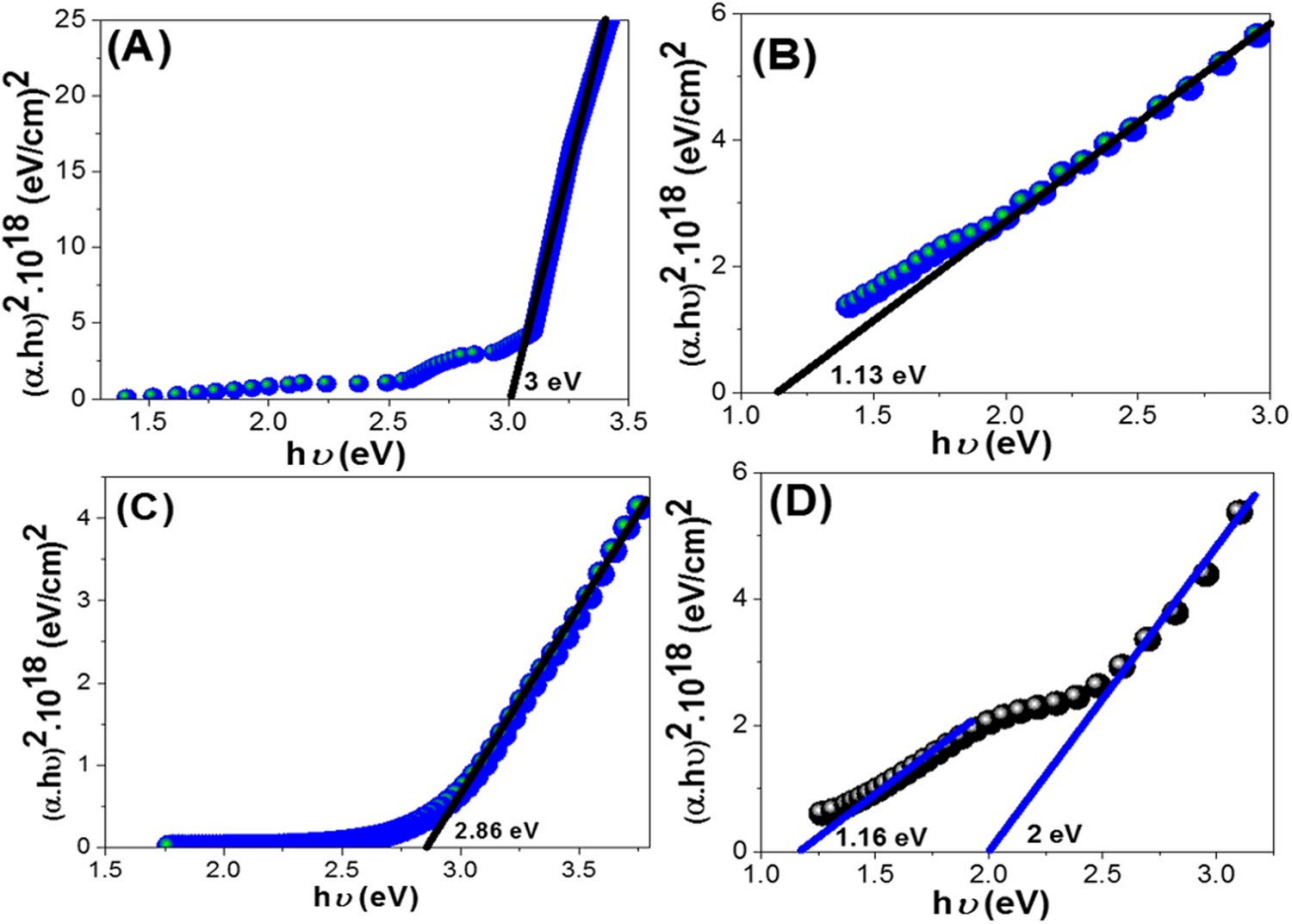
The calculated band gap values for(**A**) PANI, (**B**) PbS, (**C**) Ro-GO, and (D) PbS/Ro-GO/PANI nano/microcomposite.

The band gap values are estimated by extrapolating the linear part of the (α h*v*)^2^ curve to intercept with the h*v* axis as shown in Fig. 6. From Fig. 6(A), the value of the band gap for PANI is 3.0 eV, which agrees well with the value that obtained by that study^47^. The band gap value for PbS is 1.13 eV, Fig. 6(B), which is very close to the value that obtained by M. Hassan^50^. The Ro-GO shows a band gap of 2.86 eV. This value agrees well with that reported by Velasco-Soto *et al*.^51^. Finally, PbS/Ro-GO/PANI nano/microcomposite has two band gaps at 1.16 and 2.0 eV. This is due to the signifying effect of the synergistic interaction of PbS, Ro-GO and PANI matrix^52^, this is confirmed through the FT-IR shift peaks and X-ray analyses. Moreover, the values of band gap clearly refer to the enhancement of the optical properties of the nano/microcomposite and its suitability for application in H_2_ generation systems.

### Photoelectrochemical H_2_ generation

The photoelectrochemical (PEC) behaviors of the membrane electrodes supported with ITO glass or sputters with ultrathin Au layer; PbS/Ro-GO/PANI/ITO and Au/PbS/Ro-GO/PANI, were measured in dark and light with and without optical filters. The PEC behaviors were measured under illumination of 400 W metal-halide Lamp in 100 ml of 0.3 M Na_2_S_2_O_3_ solution at room temperature (25 °C) with a sweep rate of 1 mV/s. The prepared nanocomposite electrode with 1 cm^2^ surface area is used as the photoanode, and Pt-electrode of the same area is used as the counter electrode. Upon exposure to light, the large surface area of the nano/microcomposite electrode will produce a high density of electron–hole pairs, which will motivate the splitting of H_2_O molecules under the effect of light to carry out the hydrogen generation reaction.

**Figure 7:**
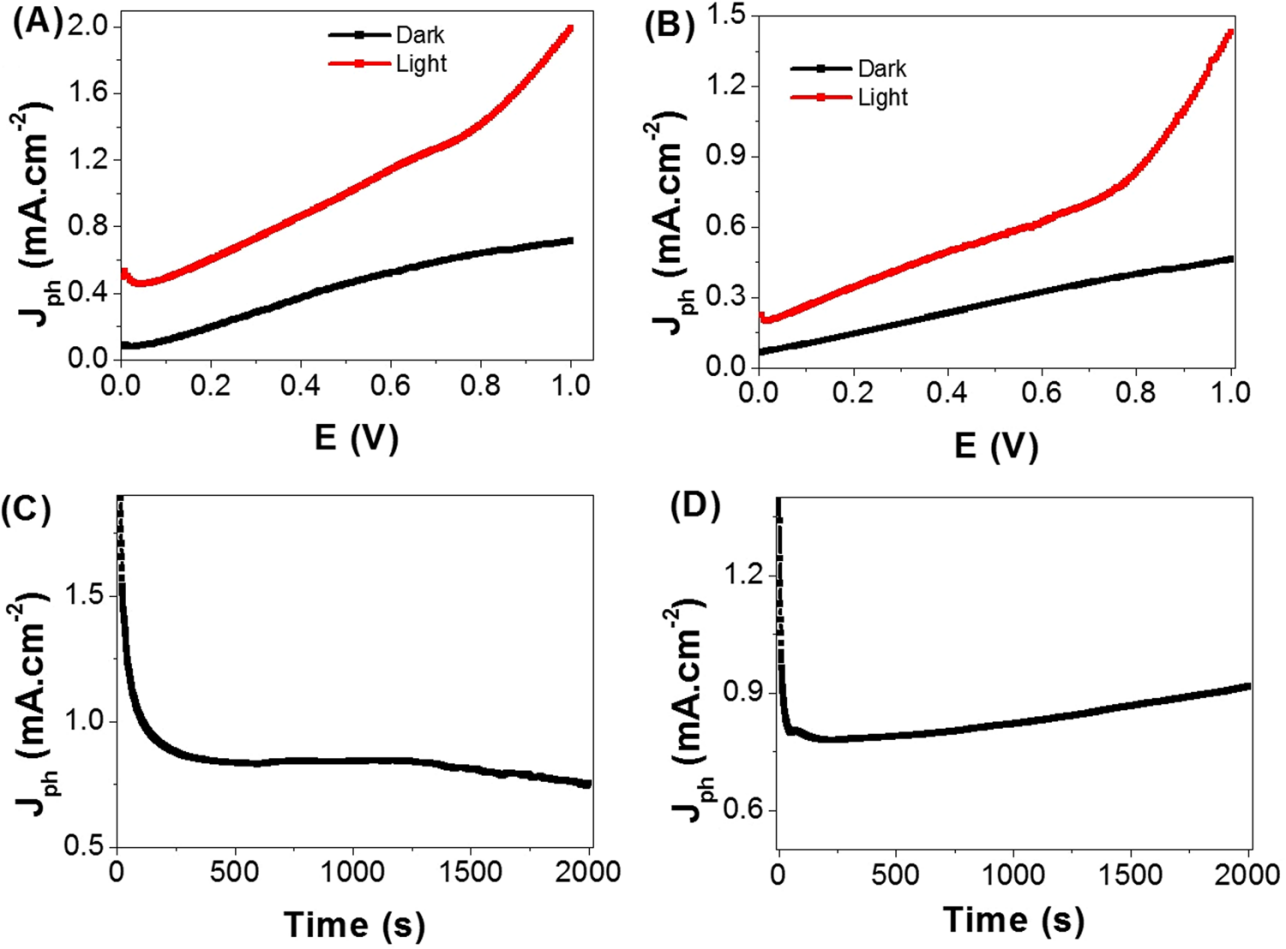
PbS/Ro-GO/PANI membrane electrodes photocurrent density-voltage curves supported on (**A**) ITO glass and (**B**) ultrathin Au layer. Current density – time characteristic of same electrode on (**C**) ITO glass and (**D**) ultrathin Au layer.

From Fig. 7(A,B), the current density-voltage (J_ph_–E) behaviors of the two electrodes are strongly affected by light exposure. For PbS/Ro-GO/PANI/ITO glass configuration, the current densities are 0.72 and 1.98 mA.cm^−2^ in dark and light, respectively. Whereas, the current densities for Au/PbS/Ro-GO/PANI electrode are 0.45 and 1.45 mA.cm^−2^ in dark and light respectively. The significant dark currents are ascribed to the charge transfer that promoted by ionic currents comes from Na_2_S_2_O_3_ source electrolyte and the existence of a secondary broad absorption band centered at ~700 nm and extended to near IR region. Under illumination, the photocurrent is generated where the oxidation reaction occurred at the photoanode and the reduction reaction at counter electrode. As shown from Fig. 7, the photocatalytic behavior of the nano/microcomposite membrane electrodes are improved and the current density increased by increasing the applied voltage. Also, the values of the current density of the electrodes are firmly affected by the supporting materials that act as a current collector. I.e., the effect of ITO glass is more than the ultrathin Au layer.

To study the effect of each component in the electrode, the photocurrent density-voltage curves of PANI/ITO, GO/ITO, and PbS/ITO electrodes in the dark and under artificial light illumination are measured and presented in Fig. S1(A,B,C), respectively. From Fig. S1(A), PANI/ITO electrode is slightly affected by light and acts as a photocathode, in which the values of the J_ph_ in dark and light at −1 V are −0.061 and −0.066 mA.cm^−2^, respectively. PANI consists of mobile free electrons responsible for conductivity with asymmetry nature. Matveeva *et al*.^53^ discussed the charge transfer behavior of PANI/ITO interface. The protonation of the ITO surface introduces some sort of charge exchange sites or current passes that reduce the additional barrier for charge transfer processes on the ITO/PANI interface and make easier charge transfer through them.

From Fig. S1(B,C), both GO/ITO and PbS/ITO electrodes work as photoanodes, in which the J_ph_ values in light at 1 V are 0.53 and 0.78 mA.cm^−2^, respectively. Compared to these values, our proposed configurations showed enhanced current densities 1.98 and 1.45 mA.cm^−2^ in light for PbS/Ro-GO/PANI/ITO and Au/PbS/Ro-GO/PANI electrodes, respectively, (Fig. 7). These results agree well with the optical properties that discussed in Fig. 5. This enhancement may be ascribed to the rules of the used components on the composite. PbS nanomaterials acts as the main photocatalyst material for H_2_ generation process, from which most of the photoelectrons produced^21^. GO nanomaterials can produce photoelectrons under the effect of light, but its main role is electron acceptor for the final H_2_ production process. Moreover, GO are supporting matrix for transports the photoelectrons produced by PANI and PbS nanoparticles and improves the charge separation process^54^. In addition, Au nanomaterials is an attractive metal with a localized surface plasmon resonance in the visible region of the electromagnetic spectrum^55^. Due to the absorption of a photon at the plasmon resonance frequency, there is a coherent oscillation in the free electrons occur, and so a high electric field near the metal nanoparticle is formed. For the oscillator in Au nanoparticles (localized surface plasmon) that couple with the oscillator in the composite nanoparticles and produce an electron-hole pair, the resonant frequencies are the same^56^. From this composite, the Au nanoparticle works as an antenna that absorbs the light, and the composite nanoparticles work as a reaction center that can promote the photochemistry (water splitting) for the H_2_ generation.

The variations of J_ph_ values with the electric potential of the PbS/Ro-GO/PANI/ITO and Au/PbS/Ro-GO/PANI electrodes configurations under the illumination of monochromatic light are mentioned in Fig. S2. The optical filters of different wavelengths from 390 to 636 nm are used to control the wavelength of the illumination at 25 °C. From this figure, the white light shows the highest J_ph_ values for the two nano/microcomposite membrane electrodes configurations. But the electrode configuration on ITO has more values than the sputtered electrode with ultrathin Au layer. By using the optical filters, the J_ph_ values decrease with increasing the optical wavelength from 390 to 508 nm, then increase with the wavelength of 587 nm and decrease again at 636 nm. Then, the maximum J_ph_ values are obtained at wavelengths of 390 and 587 nm, which matched with the absorption peak positions (Fig. 5(C). Also, the distinct behavior of the photoanodes can be tentatively attributed to the enhanced solar absorption by the PbS/Ro-GO/PANI nano/microcomposite that can cover a large portion of the solar spectrum.

The stability of the PbS/Ro-GO/PANI/ITO glass and Au/PbS/Ro-GO/PANI nano/microcomposite electrodes is investigated for a prolonged time and shown in Fig. 7(C,D), respectively. During these experiments, a small bias voltage of 0.75 V is applied between the photoanode and the counter electrode to overcome any external losses of the measuring system. Current density-time measurement curves initially show fast decrease in current density and attain a saturation value after appropriate period. The decrease of the current density until a minimum is reached indicating the continuous accumulation of uncompensated ionic space charge at the two electrodes until point electronic charge injection begins^57^. From Fig. 7(C), the J_ph_ values are decreased sharply from 1.98 to 0.86 mA.cm^−2^ as the time increased to 260 s. By increasing the time from 260 to 1500 s, J_ph_ values almost remains constant due to the increasing accumulation of the ionic charges, which suggests a longer lifetime of the PbS/Ro-GO/PANI/ITO composite electrode. Also, with increasing the time to 2000s, the high density of surface states may lead to a significant pinning of the Fermi level that can facilitate the participation of these defect states in the surface oxidation process, leading to small degradation of the composite electrode^58^. From the relation between J_ph_ and change in times, this configuration is appropriate to work in the PEC H_2_ generation experiment with a lifetime efficiency of 43.4% for 2 h.

In the case of Au/PbS/Ro-GO/PANI nano/microcomposite electrode (Fig. 7(D)), similar behavior is observed as in Fig. 7(C), but the J_ph_ values are decreased more sharply during only 60 s to 0.79 mA.cm^−2^. Also, J_ph_ almost remains constant up to 1000 s and then its value slightly increased due to the accumulation of the ionic charges up to 2000s. From the relation between J_ph_ and change in times, the electrode has high stability for PEC H_2_ generation with a lifetime efficiency of 64.2% for 2 h^58^. This higher stability is a result of the Au coating layer that covers and protects the surface of the electrode for a long lifetime, in addition to its surface plasmon resonance role for enhancing the light absorbance.

**Figure 8:**
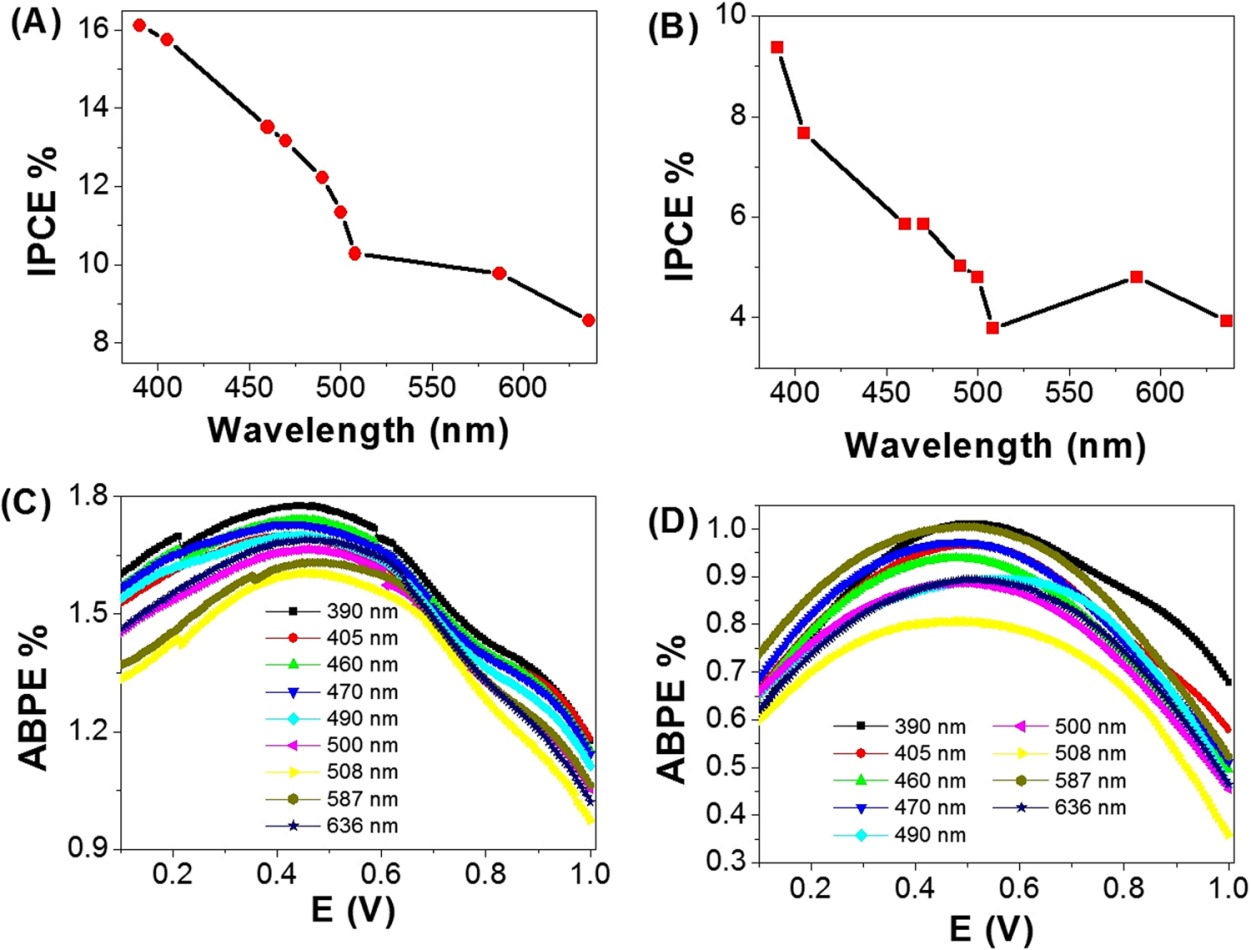
IPCE % and ABPE % as a function of wavelength for (**A**,**C**) PbS/Ro-GO/PANI/ITO glass and (**B**,**D**) Au/PbS/Ro-GO/PANI electrode configurations, respectively.

The enhanced IPEC properties of the two PbS/Ro-GO/PANI/ITO glass and Au/PbS/Ro-GO/PANI nano/microcomposite membrane electrodes are further confirmed by measuring the incident photon-to-current conversion efficiency (IPCE) under monochromatic illumination conditions as shown in Fig. 8(A,B), respectively. Such analytical measurements can also give a meaningful insight into the contribution of PbS/Ro-GO/PANI nano/microcomposite in the conversion of the incident photons into charge carriers. The IPCE is determined at an applied potential of 1 V from Eq. (3)^59^:

3 $$\mathrm{IPCE}\,( \% )=1240\,.\,\frac{{{\rm{J}}}_{{\rm{ph}}}}{{\rm{\lambda }}\,.{\rm{\rho }}}\,.\,100$$

Where λ is the wavelength of the illuminating monochromatic photons and ρ is the illuminating light power density (mW.cm^−2^). From Fig. 8, the two electrode configurations show similar IPCE% behaviors versus the wavelength of the incident photons, but the PbS/Ro-GO/PANI/ITO glass configuration shows higher IPCE values than Au/PbS/Ro-GO/PANI configuration. Based on the optical behavior of the nanocomposite, Fig. 5(C), two optimum values for IPCE% are obtained at 390 and 587 nm. For PbS/Ro-GO/PANI/ITO glass, Fig. 8(A), the optimum IPCE is 16.17% @ 390 nm. While in the case of Au/PbS/Ro-GO/PANI, Fig. 8(B), the optimum IPCE value is 9.4% @ 390 nm. The values of IPCE at 587 nm are 9.79 and 4.88% for PbS/Ro-GO/PANI/ITO and Au/PbS/Ro-GO/PANI, respectively.

To fully evaluate the PEC performance of the two electrode configurations, we calculated the applied bias photon-to-current efficiency (ABPE). ABPE represents the development of the photoelectrode performance as a function of the applied potential. The ABPE efficiency values for the designed photoelectrodes are calculated by using Eq. 4 ^59^:

4 $$\mathrm{ABPE}\,( \% )={{\rm{J}}}_{{\rm{ph}}}\frac{(1.23-{{\rm{V}}}_{{\rm{app}}})}{\rho }\,.\,100$$

where Jph is the measured photocurrent density, 1.23 is the standard state reversible potential of H_2_O, V_app_ is the applied potential during the measurement of the photocurrent density.

For PbS/Ro-GO/PANI/ITO configuration, Fig. 8(C), as the applied potential increases, the ABPE % attains its maximum value at potential value 0.58 V and wavelength 390 nm and then decreases again when the applied potential is approaching to the thermodynamic H_2_O potential (1.23 V). As the wavelengths of the incident photons increase from 390 to 508 nm, the maximum values of ABPE are decreased from 1.74 to 1.61% and its position is shifted from 0.58 to 0.43 V. Moreover, with further increasing of the wavelengths to 636, the ABPE % increase again to 1.69% with potential value of 0.45 V. In the case of the electrode Au/PbS/Ro-GO/PANI as shown in Fig. 8(D), the electrode has almost the same behavior of the previous electrode, in which as the ABPE % attains its maximum value at 0.50 V and 390 nm, and then decreases again when the applied potential is approaching to the thermodynamic H_2_O potential (1.23 V). With increasing of the wavelengths from 390 to 508 nm, the ABPE % decreases from 1.01 to 0.80% and the potential position shifts from 0.48 to 0.50 V. with further increasing of the wavelengths to 636 nm, the ABPE % increase again to 0.89% at a potential of 0.51 V. For comparison, the obtained values of IPCE, ABPE, and J_ph_ of the present study and previously reported values of relevant or similar materials are shown in Table 2 ^26–30,60–66^. The reported values in this study for IPCE and ABPE are higher than that previously obtained for the displayed materials or composites in Table 2.

**Table 2:** Comparison of IPCE and ABPE values of the present work with previously reported values for relevant or similar materials or composites.

Electrodes materials and references	Electrolyte	IPCE % (390 nm)	ABPE %	J_ph_ (mA.cm^−2^)
Tungsten disulfide/poly(3,4-ethylenedioxythiophene)/Au^26^	H_2_SO_4_	—	—	−1.4 to −2.6
Poly(3-aminobenzoic acid) frame^27^	H_2_SO_4_	—	—	0.13
TiO_2_/CdS/PbS^28^	Na_2_S/Na_2_S_2_O_3_	4	—	2
PbS/TiO_2_^29^	Na_2_S/Na_2_S_2_O_3_	13	—	1.5
Au/Fe_2_O_3_^30^	NaOH	—	1	1.2
InGaN/GaN nanowires^60^	HCL	6	0.21	1.9
CdS/TiO_2_^61^	Na_2_S/Na_2_S_2_O_3_	12.9	—	6
GaN^62^	HBr	8	0.3	0.6
CuWO_4_^63^	Na_2_CO_3_/NaHCO_3_	8	—	0.5
ZnO/TiO_2_/FeOOH^64^	Na_2_S_2_O_3_	—	0.36	1.59
Co-Pi/TiO_2_/C_3_N_4_^65^	Na_2_S_2_O_3_	—	—	1.6
SnO_2_/TiO_2_^66^	Na_2_S_2_O_3_	—	—	0.4
Au/PbS/Ro-GO/PANI	PresentWork	Na_2_S_2_O_3_	9.4	1.01
PbS/Ro-GO/PANI/ITO			16.17	1.75

For the experimental study, the reproducible studies are very important for confirming the obtained data^67^. Then, the statistical analysis of the J_ph_ – E curves for the PbS/Ro-GO/PANI/ITO and Au/PbS/Ro-GO/PANI electrodes under illumination of 400 W metal-halide lamp without the optical filter are carried out and displayed in Table 1S. These data are calculated depending on the reproducible studies of J_ph_ – E curves for the two electrodes under light irradiation (Fig. S3). From Fig. S1 and Table S1, the J_ph_ values for the PbS/Ro-GO/PANI/ITO electrode are measured three times with Relative Standard Deviation (RSD) of 2.7% with a mean value of 1.99 mA.cm^−2^, respectively. While Au/PbS/Ro-GO/PANI electrode has RSD of 2% and a mean value of 1.44 mA.cm^−2^.

Finally, the mechanism of H_2_ generation from H_2_O using the PbS/Ro-GO/PANI electrode is shown in Fig. 4S. The PANI and RO-GO materials act as a supporting surface to PbS nanoparticles, in which the size of PbS is very small in comparison with them. Under the effect of artificial light, the levels of PANI are split, in which the electrons excitations take place. The transfer of electrons occurs from LUMO to HOMO levels^68^. Because of the existence of the potential difference between PANI and PbS levels, the HOMO electrons of PANI inject to conducting band (CB) of PbS nanoparticle. Then, the PbS nanoparticles serve as electron donor material, in which the excited electrons can transfer directly to H_2_O or to the Ro-GO material that acts as a current collector, then from which the electrons can transfer to H_2_O for H_2_ production process^21^. In the other side, the Na_2_S_2_O_3_ (sacrificing agent) accept the holes from PANI for the O_2_ evolution with the help of OH^−^ radicals^68^. This electron-hole transition process is repeated with the lifetime of the prepared nanocomposite electrode that indicates a photocatalytic activity of the photocatalyst powder^69^.

## Conclusion

Rolling graphene oxide (Ro-GO), polyaniline (PANI) nano/microparticles, and PbS nanoparticles have been successfully fabricated by modified Hammer, oxidative polymerization, and chemical bath deposition methods, respectively. PbS/Ro-GO/PANI nano/microcomposites were designed using different weights of PbS  and Ro-GO and their optical properties were studied using UV-Vis spectrophotometer. Based on the optical properties, the structural and morphological properties of the optimized (0.075 g)PbS/(0.125 g)Ro-GO/(0.4 g)PANI nano/microcomposite and its individual constituents were studied using FTIR, XRD, SEM, and HR-TEM. From the optictal properties study, band gaps of 3, 1.13, 2.86, and (1.16, 2) eV were observed for PANI, PbS, Ro-GO, and PbS/Ro-GO/PANI composite, respectively. Using the optimized ratios, two photoelectrode configurations (Au/PbS/Ro-GO/PANI and PbS/Ro-GO/PANI/ITO/glass) were assembled by the sputtering the first with 45 nm Au and glued the second on ITO glass substrate. These two configurations were applied for the PEC hydrogen generation and their performances were evaluated using I-V and I-t characteristics. Also, the IPCE and ABPE efficiency were calculated under monochromatic illumination conditions. The lifetime efficiency values were 64.2 and 43.4% for the first and second electrode for 2 h measurement, respectively. The values of IPCE and ABPE at 390 nm were (9.4% and 1.01%) for the first configuration, and (16.17%, 1.75%) for the second configuration.

## Methods

### Preparation of PANI, Ro-GO, PbS and PbS/Ro-GO/PANI nano/microparticles

PANI is prepared by polymerization method using rapid mixing technique, in which 0.1 M aniline (Rankem company, India, 99%) is dissolved in 0.5 M HCl (El Nasr chemical company, Egypt, 99%) under ultrasonication for 15 min at 298 K. 0.15 M (NH_4_)_2_S_2_O_8_ (Winlab company, UK, 99.2%) is prepared by the same method and added over the dissolved aniline suddenly. The formation of the green precipitate refers to the polymerization of aniline to PANI. Then, PANI is washed several times with warm water.

The preparation of GO is carried out by the modified Hummers method^70^. 1 g of graphite powder (Alpha Chemika, Mumbai, 99.9%) is added to a mixture of 120 ml H_2_SO_4_ (El Nasr chemical company, Egypt, 99%) and 14 ml H_3_PO_4_ (BioChem company, Egypt, 99.8%). The process takes place in an ice bath. Then 6 g of KMnO4 is added slowly to the mixture under magnetic stirring for 1 h. The mixture is left for 24 h over the magnetic stirrer at 50 °C. Then, 800 ml of (0.05 M) H_2_O_2_ (BioChem company, Egypt, 99.8%) is added drop by drop to the mixture in an ice bath to reduce the residual of the KMnO_4_ (El Nasr chemical company, Egypt, 99%). The mixture releases a lot of bubbles and the color of the mixture changes into brilliant yellow. For further purification, the as-prepared graphene oxide is re-dispersed in DI water and then is dialyzed for one week to remove any residual salts and acids. The prepared GO nanosheets have a concentration of 11 mg/ml. To obtain Ro-GO from GO, the GO nanosheets were left to stand for 2 months at room temperature in N_2_ atmospheric pressure. During this period, GO sheet is rolled to form Ro-GO nano/microparticles. The technique of this process may occur depended on the first rolled one sheet, that acts as nuclei for the other sheets to roll around it, the TEM analysis show this process.

PbS nanoparticles were prepared from 0.1 M Pb(NO_3_)_2_ (Oxford Laboratory, India, 99%) and 0.1 M Na_2_S (Alpha Chemika, Mumbai, 99.9%) solutions at 298 K. Pb(NO_3_)_2_ and Na_2_S solutions are ultrasonicated for 15 min. Then, the Na_2_S solution is purred over Pb(NO_3_)_2_ and ultrasonicated for 1 h to form a black precipitate, which indicates the formation of PbS particles. Then, the black precipitate is placed in the microwave for 15 minutes in the N_2_ gas atmosphere. Finally, the prepared nanoparticles are washed well with warm water several times and dried at 60 °C for 24 h.

The PbS/Ro-GO/PANI nano/microcomposite was developed using PANI, Ro-GO, PbS nanostructures. The ratios of Ro-GO, PbS in the composite were optimized based on the optical properties of the composite. For optimizing the ratio of PbS, 0.4 g of PANI nano/microparticles was ultrasonicated with 2.25 ml (0.025 g) of Ro-GO for about 30 min and then mixed with different concentrations of Pb(NO_3_)_2_ solutions (0.0001 to 0.0007 M). Na_2_S solutions were poured over the solutions to precipitate PbS nanoparticles with different weight (from 0.025 to 0.175 g). For optimizing the Ro-GO ratio, 0.4 g PANI is ultrasonicated with various weights of Ro-GO nano/microparticles (0.025 to 0.175 g) for 30 minutes, then mixed with the optimized concentration of Pb(NO_3_)_2_. Na_2_S solution is added suddenly to precipitate the PbS over Ro-GO/PANI composite. Finally, the PbS/Ro-GO/PANI composite is collected and washed well with distilled water several times and dried at 60 °C for 24 h.

### Preparation of two electrode configurations utilizing PbS/Ro-GO/PANI nano/microcomposite

Two electrode configurations were prepared using the optimized PbS/Ro-GO/PANI nano/microcomposite and applied for the photoelectrochemical H_2_ generation. In both cases, 3% of the composite was mixed with 3% dibutyl phthalate (DBP) (Middle-east company, Egypt, 99.8%) and 3% polyvilny chloride (PVC) (Middle-east company, Egypt, 99.8%). All the components were mixed well and dissolved in minimum volume of tetrahydrofuran (THF) (Middle-east company, Egypt, 99.9%). The resulting mixture was transferred into a Petri dish of 5 cm diameter. The total weight of constituents in each batch was fixed at 0.35 g. The Petri dish was then covered with a filter paper and left to dry in air. To obtain a uniform electrode thickness, the amount of THF was kept constant, and its evaporation was fixed for 24 h. The thickness of the electrode is ~0.2 mm. 10 mm diameter disk was cut out from the prepared electrode and glued to one side of ITO glass slide (Aldrich, 20 Ω) using Ag-THF paste. Another disc is coated with Au layer of 45 nm thickness using sputter coating technique at pressure 2 Torr and distance 8 cm in front of the Au target (99.99%).

### Nano/microparticles characterization

The characterizations of the prepared nanostructures and nano/microcomposite were studied using high-resolution X-ray diffractometer system (model: PANalytical X’Pert Pro, Holland) with CuK ${\rm{\alpha }}$ radiation (${\rm{\lambda }}$ = 1.5406 A°), operated at 45 kV and 40 mA. The XRD patterns were recorded in the 2θ range 10–90°. The pattern was analyzed by matching the observed peaks with the standard patterns provided by JCPDS files. Also, scanning analyses were carried out using Scanning Electron Microscope, SEM, (Model: ZEISS SUPRA 55 VP and ZEISS LEO, Gemini Column), and Transmission Electron Microscope, TEM, (JEOL JEM-2100 TEM). The UV-visible absorption spectra of the prepared structures were measured using Shimadzu UV spectrophotometer (M160 PC) at room temperature in the range 200–1100 nm. Fourier transform infrared spectroscopy (FTIR) measurements were carried out using Shimadzu FTIR-340 Jasco spectrophotometer.

### Photoelectrochemical H_2_ generation test

The photocatalytic hydrogen electro-generation experiments were performed by two nano/microcomposite electrodes supported on Au and ITO glass. The photoelectrochemical current-voltage (I-V) and the current-time (I-T) behaviors were measured using Keithley measurement – source unit (2400 SourceMeter, A Tektronix company). Nano/microcomposite electrodes (1 cm^2^) used as a working electrode, while Pt-electrode with the same dimensions was used as a counter electrode. 100 ml of 0.3 M Na_2_S_2_O_3_ was used as the source electrolyte. The cell was exposed to an artificial light lamp (blended metal halide lamp 400 W, China) provided with series of linear optical filters.

## Electronic supplementary material

Supplementary information
