# Supplementary material for: Photocatalytic properties of PbS/graphene oxide/polyaniline electrode for hydrogen generation
Source: Sci Rep. 2017 Oct 26;7:14100. doi: 10.1038/s41598-017-14582-8 (PMC5658379; doi:10.1038/s41598-017-14582-8)
Supplement: Supplementary file 1 — Supplementary information [file 41598_2017_14582_MOESM1_ESM.pdf]

Supplementary information

Photocatalytic properties of PbS/graphene oxide/polyaniline electrode for hydrogen generation

Mohamed Shaban[1], Mohamed Rabia[1,2,*], Asmaa M. Abd El-Sayed[1], Aya Ahmed[1], Somaya Sayed[1],

[1] Nanophotonics and Applications (NPA) Lab, Department of Physics, Faculty of Science, Beni -Suef University, Beni-Suef, 62514, Egypt

[2] Polymer Research Laboratory, Chemistry Department, Faculty of Science, Beni-Suef University, Beni-Suef 62514, Egypt.

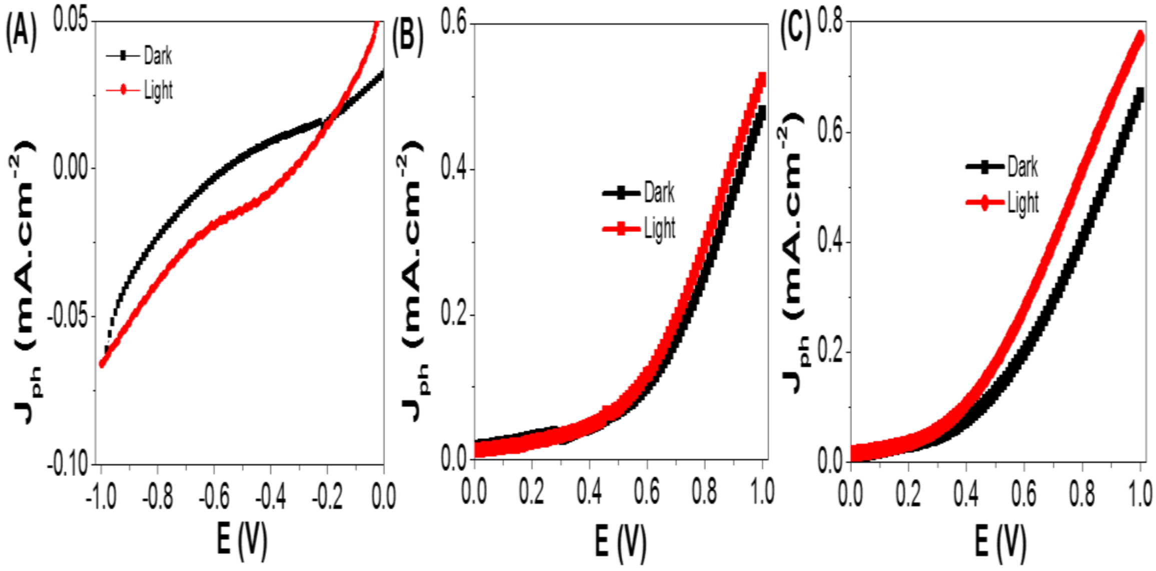

Figure 1S **Photocurrent density-voltage curves of (A)PANI/ITO, (B)GO/ITO, and (C) PbS/ITO electrodes in the dark and light under illumination of metal-halide Lamp without the optical filter.**

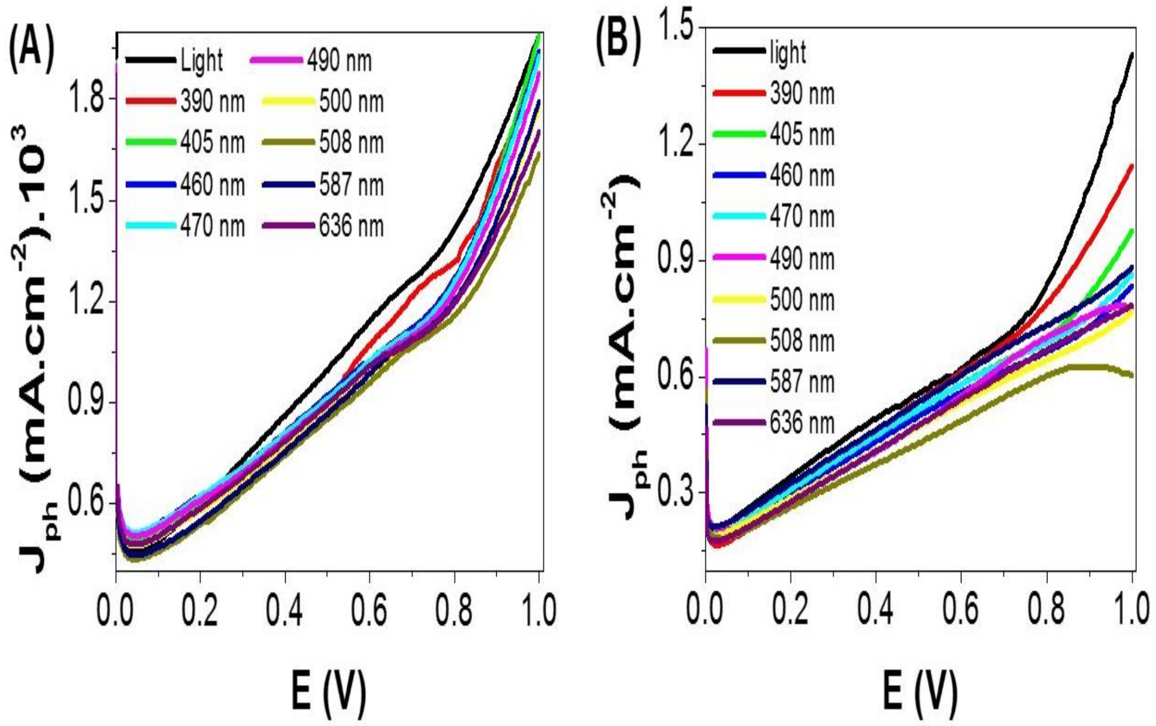

Figure 2S **J_{ph} - E behaviors of the nano/microcomposite membrane electrodes supported on (A) ITO glass and (B) Au thin film under monochromatic light illumination using optical filters of different wavelengths.**

Table 1S **Statistical analysis for J_{ph}-E curves of the PbS/Ro-GO/PANI/ITO and Au/PbS/Ro-GO/PANI electrodes under illumination of metal-halide Lamp without the optical filter.**

**Configuration**	**Mean**	**Standard deviation %**	**Relative standard deviation %**	**Minimum**	**Maximum**
**PbS/Ro-GO/PANI /ITO**	1.99	4.7	2.7	1.96	2.05
**Au/PbS/Ro-GO/PANI**	1.44	3.6	2.0	1.4	1.47

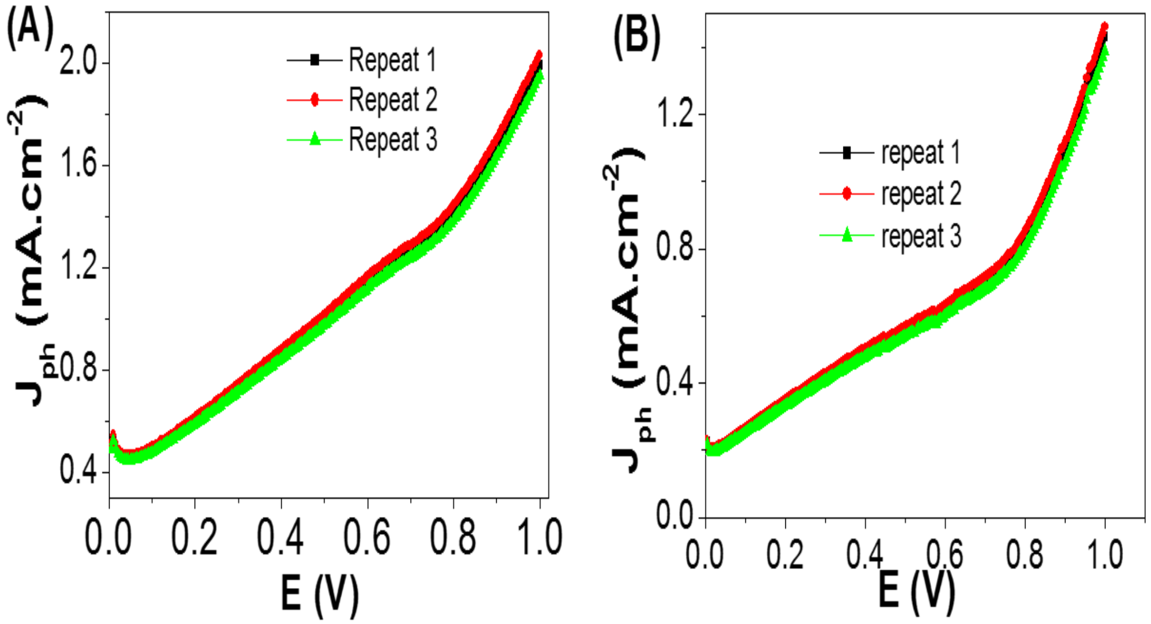

Figure 3S **Reproducible studies of photocurrent density-voltage curves of PbS/Ro-GO/PANI membrane electrodes (A) on ITO glass and (B) sputtered with ultrathin Au layer under illumination of 400 W metal-halide Lamp without the optical filter.**

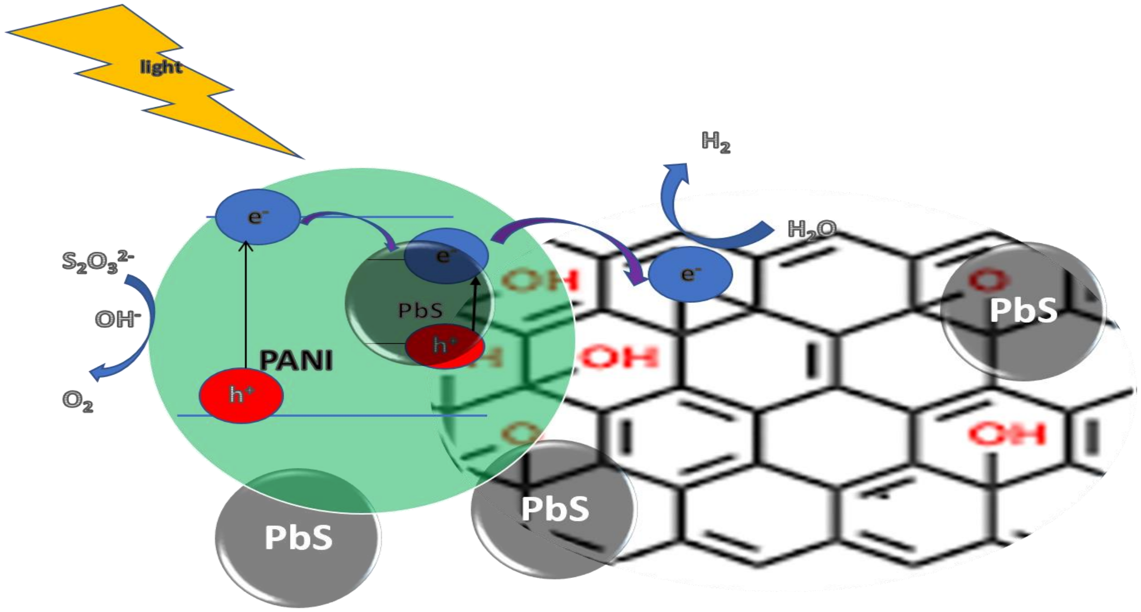

Figure 4S **The mechanism of H_2 generation using the prepared composite electrodes PbS/Ro-GO/PANI.**
